# A Novel Assay for Screening Inhibitors Targeting HIV Integrase LEDGF/p75 Interaction Based on Ni^2+^ Coated Magnetic Agarose Beads

**DOI:** 10.1038/srep33477

**Published:** 2016-09-16

**Authors:** Zhang Dawei, He Hongqiu, Liu Mengmeng, Meng Zhixia, Guo Shunxing

**Affiliations:** 1 Institute of Medicinal Plant Development, Chinese Academy of Medical Sciences & Peking Union Medical College, Beijing, 100193, China; 2 Institute of Bioinformatics and Medical Engineering, School of Electrical and Information Engineering, Jiangsu University of Technology, Changzhou, 213001, China; 3 Chongqing Center for Biomedicines and Medical Equipment, Chongqing Academy of Science and Technology, Chongqing, 401123, China

## Abstract

HIV-1 integrase (IN) plays an essential role in viral replication and thus serves as an important target for chemotherapeutic intervention against HIV-1 infection. However, the current three clinical IN inhibitors, raltegravir, elvitegravir and dolutegravir share the same inhibitory mechanism, resulting in a common clinical resistance profile which have emerged in infected patients receiving treatment. Therefore, it is important to develop small molecule inhibitors that impair IN function with distinct mechanisms of action. In this work, a magnetic-beads based biochemical assay targeting the protein-protein interaction (PPI) between HIV IN and the cellular cofactor LEDGF/p75 was developed for identification of HIV-1 IN inhibitors. Furthermore, a library containing 1000 US. Food and Drug Administration (FDA)-approved drugs currently used for human medication was screened to identify inhibitors targeting the PPI. The assay was proved to be quite robust and with the novel assay we successfully identified dexlansoprazole (IC_50_ of 4.8 μM), a FDA-approved proton pump inhibitor, as a potential inhibitor for the PPI between IN and LEDGF/p75, which bound to the LEDGF/p75 partner with a kinetic dissociation (Kd) constant of 330 nM ± 2.6 nM.

Human immunodeficiency virus type 1 (HIV-1) integrase (IN) is a critical enzyme in the virus replication cycle as it is required for the covalent integration of a double-stranded DNA copy of the viral RNA genome into the infected host cell chromosomes^1^. IN mainly mediates two spatially and temporally separated and energetically independent reactions, the 3′ processing and the strand transfer reaction. IN has been validated as a therapeutic target for anti-retroviral drug development^2^. Currently, there are three FDA-approved integrase inhibitors: raltegravir (RAL), elvitegravir (EVG), and dolutegravir (DTG) in clinical usage. These inhibitors all impair strand transfer activity of IN and are collectively termed as IN strand transfer inhibitors (INSTIs)^3^. Recent study suggests that inhibition of integration will not only block viral replication, but should also enhance T cell survival^4^,^5^,^6^. Despite of the achievements in the development of the INSTI class, drug-resistant (and multidrug-resistant) HIV-1 strains to this inhibitor class can evolve readily in the clinic. In fact, drug-resistant mutations to all three clinically available INSTIs have emerged in infected patients receiving treatment^7^,^8^,^9^,^10^. Therefore, it is of great importance to develop novel inhibitors that target IN out of its active site while overcoming INSTI resistant mutants.

The viral replication of HIV-1 depends on cellular co-factors and cellular pathway^11^. Host cell proteins that particularly assist the viral integration have been determined and termed as integration cofactors (INCFs). The INCFs have been proposed to play a role in one of the following steps: catalysis, nuclear import, target site selection, or repair of the single-stranded DNA gaps that occur at viral/chromosomal DNA junctions during integration^12^. Among these identified INCFs, lens epithelium-derived growth factor (LEDGF/p75) is the first identified and the best characterized one to date^13^,^14^. The interface of protein-protein interaction between LEDGF/p75 and HIV IN is primarily formed by HIV IN binding domain (LEDGF/p75 IBD) in the C-terminal region of LEDGF/p75 with the HIV IN catalytic core domain (IN CCD). The PPI has been validated for an effective drug target^15^,^16^, which small molecules have recently been developed to block^17^,^18^,^19^,^20^.

Up to date, assays for screening compounds that block PPI between IN and LEDGF/p75 could be classified into two main categories: (i) cellular-level screening method based on yeast two-hybrid assay^21^, and (ii) molecular-level screening methods based on Amplified Luminescence Proximity Homogeneous Assay (Alpha) or Homogeneous Time-Resolved Fluorescence^22^,^23^. However, these above assays have limitations. Yeast two-hybrid assay is low-throughput, high false positive and time-consuming, while molecular-level screening methods need expensive and sophisticated instruments which are not available to all laboratories.

It has been suggested that novel therapeutics for many diseases including infectious disease may be found by exploiting medications that are already approved for use^24^. Several precedents for compound “repurposing” exist, such as sildenafil, thalidomide and plerixafor^25^. Moreover, many approved drugs have a well-established history of safe dosing in broad populations, novel repurposing indications can likely be rapidly tested directly in human subjects, with no need of extensive preliminary safety assessments. Therefore, drug repurposing has become a mainstream strategy in drug development.

In the present study, we developed a novel magnetic beads based assay to detect the PPI between IN and LEDGF/p75. Using the assay, we undertook a drug repositioning screen to identify inhibitors of the IN–LEDGF/p75 interaction from a library of US Food and Drug Administration (FDA)-approved drugs. We found that the assay developed was quite robust. It was also found that dexlansoprazole, which is clinically used for the treatment of erosive esophagitis and gastro-esophageal reflux disease, blocked the interaction of IN–LEDGF/p75 and bound to the LEDGF/p75 partner with a kinetic dissociation (Kd) constant of 330 nM ± 2.6 nM. These results indicated that the assay we developed was effective for drug screening targeting the PPI between IN and LEDGF/p75, and dexlansoprazole may be a potential IN-LEDGF/p75 interaction inhibitor.

## Results and Discussion

A binding pocket presenting at the interface of two IN CCD monomers is an important structural feature required for PPI between HIV IN and LEDGF/p75 as a validated target for novel antivirals^14^,^26^. Previous research has proved that compounds which inhibited the PPI between the truncated forms of these two target proteins could also block PPI between the full-length proteins^22^. In this study we designed an assay targeting this binding pocket by using the truncated forms of the two target proteins, IN CCD and LEDGF/p75 IBD. The IN CCD protein used contained HIV IN residues 50–212 fused with an C-terminal His_6_-tag and incorporated the F185K mutation to improve protein solubility^27^. IN (F185K) was previously shown to interact effectively with LEDGF/p75 IBD^14^. The LEDGF/p75 IBD component used for the assay was a fusion of LEDGF/p75 residues 347–471 with the N-terminal GST-tag. All of the recombinant proteins used for assay development were affinity purified to >90% as determined by 12% SDS-PAGE (results not shown). For the PPI assay, Ni-NTA magnetic agarose beads were used to bind the PPI complex with His_6_-tag.

### Principle of the assay

**Figure 1:**
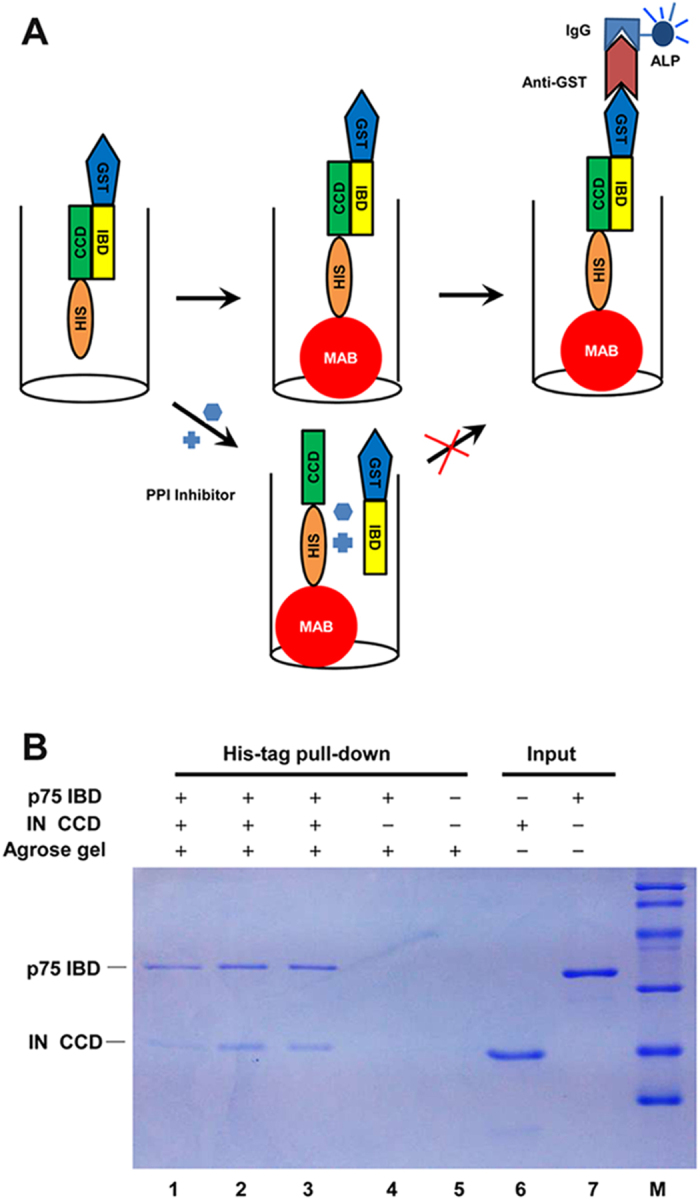
The schemes of the established assay for IN CCD-LEDGF/p75 IBD interaction and SDS-PAGE gel of His_6_-tag pull down assay for IN CCD-LEDGF/p75 IBD interaction. (**A**) GST-IBD interacts with His_6_-CCD in the microplate, forming the PPI complex which is captured by Ni^2+^-coated magnetic beads (MAB). And the amounts of PPI complex are quantified by using ALP-conjugated anti-GST antibody and ALP substrate. In the presence of the PPI inhibitors, GST-IBD cannot interact with His_6_-CCD, resulting in no ALP activity. (**B**) Recombinant LEDGF/p75 IBD was incubated with HIV-1 IN CCD containing a His_6_-tag at the C terminus (lane 1–3). The complexes were bound on a Ni^2+^ chelating resin. Lanes 6 and 7 reflect the protein input in the reactions. IN CCD was omitted in lane 4 and only agarose gel was added in lane 5. The gel was stained using Coomassie Blue R-250. The results showed that IN CCD could interact with LEDGF/p75 IBD (lane 1–3), no band was detected in controls (lane 4–5).

In the assay, GST–tagged LEDGF/p75 IBD (yellow) is mixed with His_6_-tagged IN CCD (green) at the desired concentrations. After incubation at room temperature, a double-tagged PPI complex with N-terminal GST-tag and C-terminal His_6_-tag is established between two protein partners. The PPI complex is captured by Ni^2+^-coated magnetic beads (red) through His_6_-tag, followed by the addition of alkaline phosphatase conjugated anti-GST antibody (dark red). Therefore, the product could be measured by the alkaline phosphatase conjugated-coupled enzyme reaction (Fig. 1A).

### Detection of the protein-protein interaction between LEDGF/p75 IBD and IN CCD

A His_6_-tag pull down assay was performed to validate the PPI between LEDGF/p75 IBD and IN CCD *in vitro*. The proteins in the PPI complex are identified in a SDS-PAGE gel stained with Coomassie Brilliant Blue R-250 (Fig. 1B). When 1 μg LEDGF/p75 IBD was added to 1 μg IN CCD, two bands can be identified (lane 1–3). In contrast, the negative controls indicated that without IN CCD, no band was identified. These results suggested that the recombinant LEDGF/p75 IBD and IN CCD can interact with each other *in vitro*. Previously, Busschots *et al.*^28^ has reported that compared with the C-terminal His_6_-tagged IN, N-terminal His_6_-tagged IN pulled down less LEDGF/p75 and N-terminal domain of IN enhanced the affinity of IN to LEDGF/p75. In this study, C-terminal His_6_-tagged IN CCD can pull down LEDGF/p75 IBD *in vitro*, which is concordant with previous studies by Busschots *et al.*^28^.

### Assay development and validation

**Figure 2:**
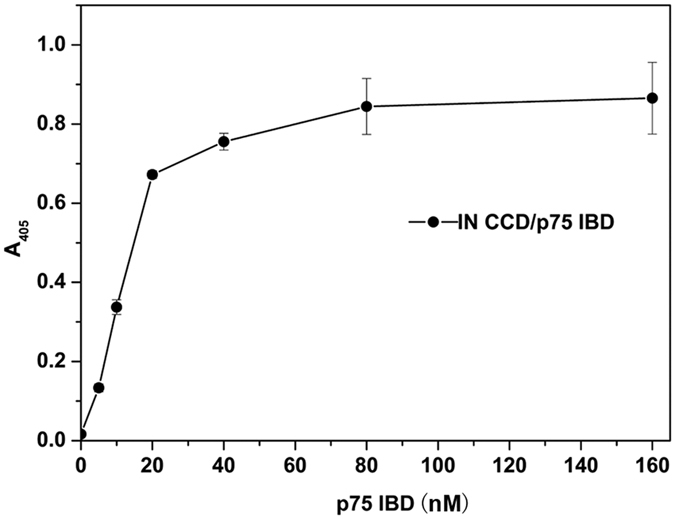
Determination of the optimal IN CCD and LEDGF/p75 IBD ratio and protein concentrations for the assay. The assay signal was measured using a fixed concentration of IN CCD (20 nM) against a titration of LEDGF/p75 IBD. Signal was obtained as A_405_. Error bars represent SD from 3 replicate values. A ratio of 1:1 IN CCD : LEDGF/p75 IBD was chosen for the subsequent assays.

This study aimed to establish a high-throughput assay for screening inhibitors targeting the PPI between IN CCD and LEDGF/p75 IBD. The optimum concentration ratio of the IN CCD and LEDGF/p75 IBD binding partners was initially determined by titrating, with increased concentrations of LEDGF/p75 IBD against a fixed concentration of 20 nM IN CCD (Fig. 2). A ratio of 1:1 IN CCD: LEDGF/p75 IBD was chosen for the subsequent assays as it was well below the binding capacity of the beads while still provided a robust signal that was within the linear range of the assay and not within the hooking zone.

**Figure 3:**
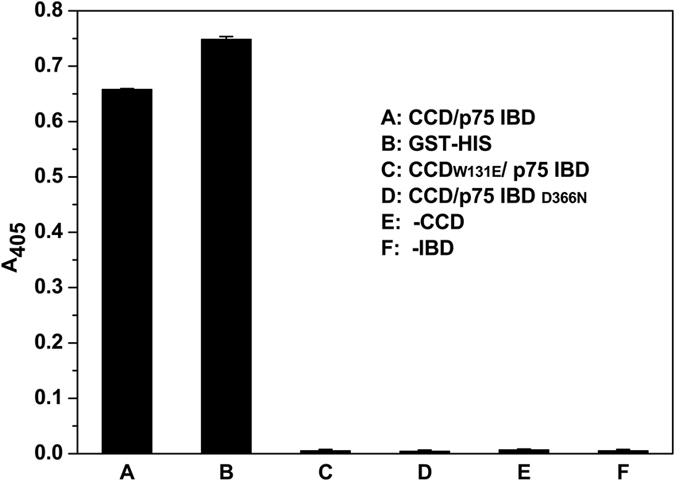
Assay performance under optimal reaction conditions. Signal was obtained as A_405_. Error bars represent SD from 3 replicate values. The S/N, S/B and z′ factor values of the assay were 326, 146 and 0.98 respective, which exceeded the minimum criteria (S/N >10, S/B >3 and z′ >0.5). These values of statistical parameters reflected that the assay developed showed high sensitivity, specificity, and robustness and was suitable to be developed for high-throughput screening (HTS).

To test the effectiveness and robustness of the assay, we measured the signal of all reactions under optimal reaction conditions. Furthermore, well-to-well variations in 96-well plates were evaluated by calculation of standard statistical parameters S/N, S/B and z′ factor. As shown in Fig. 3, the negative controls in the absence of LEDGF/p75 IBD or IN CCD and in the presence of IN CCD_W131E_-LEDGF/p75 IBD or IN CCD-LEDGF/p75 IBD_D366N_ showed background readings lower than 0.01 measured the absorbance at 405 nm (A_405_). In contrast, the positive control using the GST-His_6_ double tagged protein showed a signal of 0.76 and the IN CCD-LEDGF/p75 IBD PPI gave a signal of 0.66 at 405 nm respectively. Accordingly, the S/N, S/B and z′ factor values of the assay were 326, 146 and 0.98 respectively, which exceeded the minimum criteria (z′ >0.5, S/N >10, and S/B >3). These values of statistical parameters reflected that the assay developed showed high sensitivity, specificity, and robustness and was suitable to be applied for high-throughput screening (HTS).

**Figure 4:**
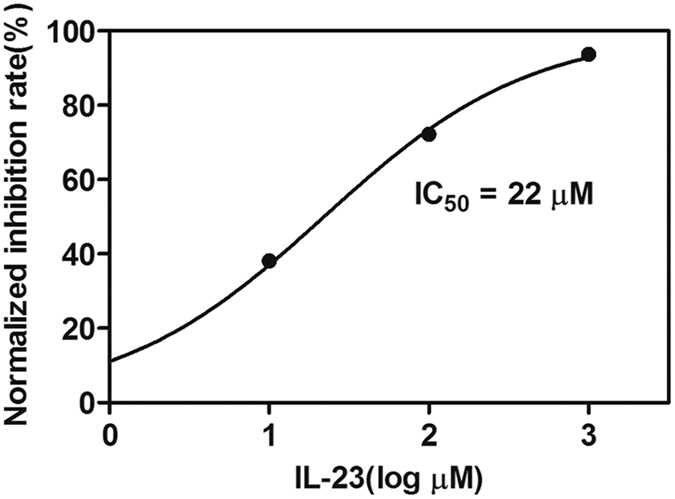
Inhibition of interaction between IN CCD and LEDGF/p75 IBD by peptide IL23. Compounds were diluted in DMSO to a final concentration of 10% (v/v) DMSO in the reaction volume. PPI binding mutant controls (single-point mutation) were set as negative controls, and the positive control contained 10% DMSO only. Error bars represent SD from 3 replicate values.

To test whether the HTS assay was effective for screening inhibitors of the PPI through detection of reduced signal, we tested optimized IN CCD-LEDGF/p75 IBD binding assay in the presence of increasing concentrations of the peptide IL-23, which was previously reported as an inhibitor of IN-LEDGF/p75 PPI^29^. As shown in Fig. 4, the assay signal decreased with the increase in IL-23 concentration, implying that the PPI was inhibited by IL-23. The IC_50_ value was calculated based on the assay results using a non-linear regression curve fit. In this assay, IL-23 inhibited the signal production with an IC_50_ of 22.0 μM (95% confidence interval [CI], 19.7 μM–24.6 μM) which was comparable to previous experiment result of 25.0 μM, indicating that the assay can robustly detect the decreases in signal and was therefore effective and suitable for a HTS application^29^.

Compared with other three screening systems currently used in HTS for IN-LEDGF/p75 interaction inhibitors^18^,^19^,^20^, the present ELISA-based assay has several improvements: (1) economic (expensive equipment and reagents are not required); (2) convenience (the value is read on an ELISA reader and could be widely used in a normal laboratory); (3) lower background (using the no-binding surface microplate), whereas the molecular-level screening methods need a fluorescence microplate reader and agents that is a bit expensive for some laboratories and a cell-based screening is not convenient for HTS.

### High-throughput screening for inhibitors of IN CCD-LEDGF/p75 IBD Interaction

**Figure 5:**
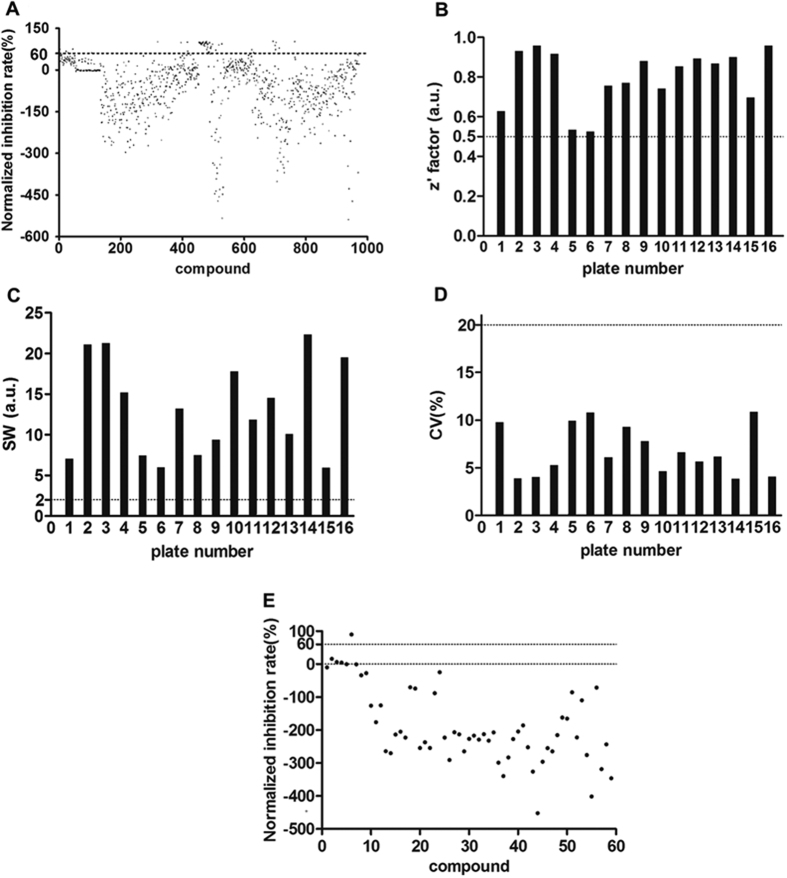
The results of HTS screening and assay quality control. (**A**) Scatterplots of the primary screening using the developed assay. Dashed line shows the “hits” selection criteria (more than 60% disruption of a PPI complex); Z′ factor (**B**), SW (**C**) and CV (**D**) of each plate in the primary screening. Dashed lines show the minimum pass criteria (z′ >0.5, S/N >10, S/B > 3, SW >2, CV < 20%). (**E**) Scatterplots of the confirmatory screening using the developed assay. Dashed line shows the “hits” selection criteria (more than 60% disruption of a PPI complex). Z′ factor, SW and CV of the confirmatory screening were 0.99, 7.32 and 14% respectively.

The optimized IN CCD-LEDGF/p75 IBD PPI assay was used to screen a partial library of pharmacologically active compounds for inhibitors of PPI. Compounds were screened as described in the materials and methods section, with appropriate comparison controls included in each plate. Hits were scored as compounds that showed an inhibition ratio of the signal higher than 60% in the assays. The z′, signal window (SW), and coefficient of variation (CV) of each plate were calculated and then compared with the minimum pass criteria (z′ >0.5, SW >2, CV < 20%) to evaluate the quality of the HTS data. As shown in Fig. 5A, at the concentration of 30 μM, 59 of the 1000 initial compounds were identified as hits from the primary screen, with more than 60% inhibition of the signal representing a hit rate of 5.9%. All HTS campaigns in the primary screening exceeded the minimum criteria (z′ >0.5, SW >2, and CV <20%) (Fig. 5B–D), indicating that the results of the screening were reliable and could be used for further investigation. These 59 primary hits were further confirmed as described in the primary screening. Among them, dexlansoprazole showed the most potent inhibitory effect with an inhibitory rate of 90.3% (Fig. 5E). All HTS campaigns in the confirmatory screening exceeded the minimum criteria (z′ >0.5, SW >2, and CV <20%), indicating that the results of the screening were reliable. Therefore, we focused our follow-up studies on dexlansoprazole.

In the primary screening, each compound was tested in one replicate (Fig. 5A). While in the confirmatory screening, each compound was used in triplicate. Therefore, results in the primary screening were less credible than that in the confirmatory screening. The repeatability of the primary 59 hits was poor as shown in Fig. 5E. Also, in the assay we developed, two kinds of beads were used, and many compounds may interact with the reaction system, leading to the nonspecific interference shown in Fig. 5A. It was reported that compounds which modulate the protein-protein interaction include two types: inhibitor and stabilizer^30^. In our manuscript, the target was LEDGF/p75-IN interaction, therefore, several compounds may stabilize the target and indicated as enhancers.

### Identification of pan-assay interference compounds

**Figure 6:**
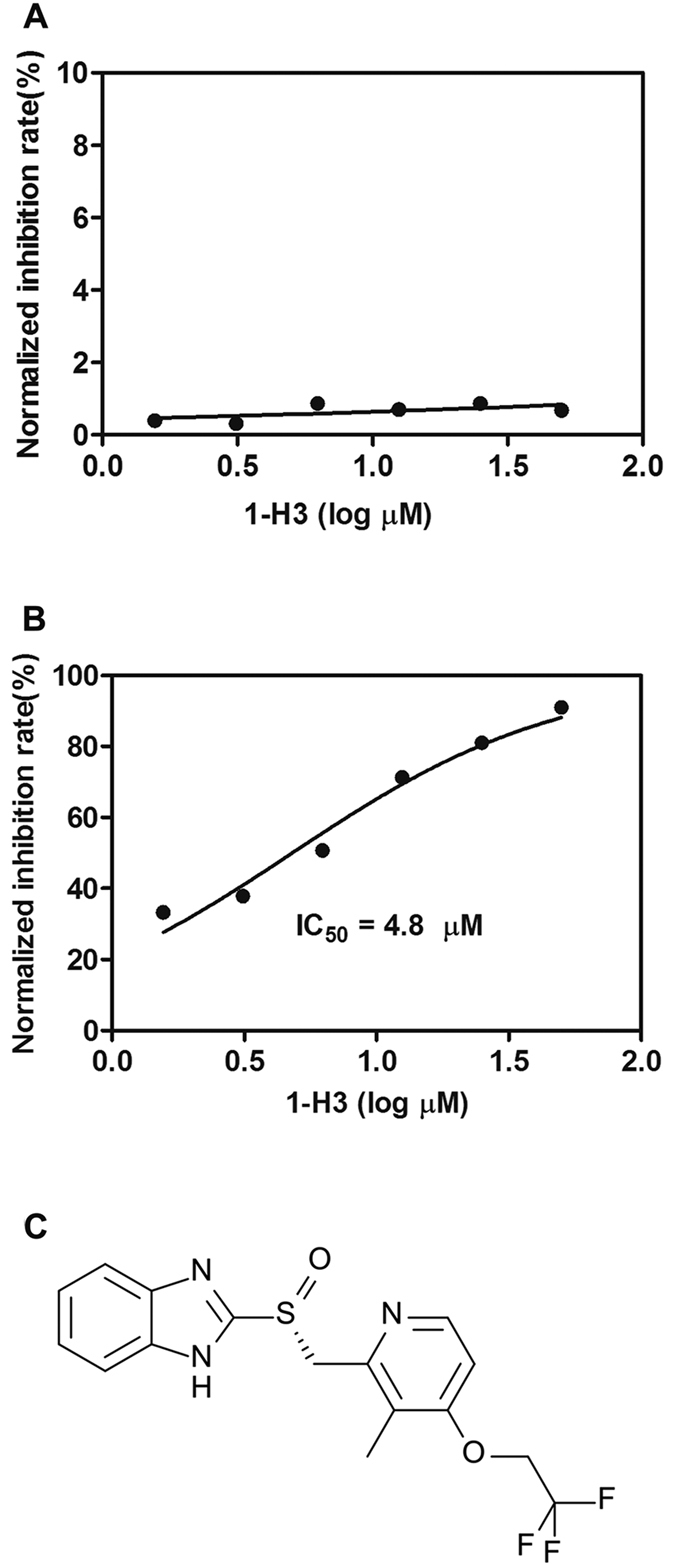
Counterscreen and dose response assay for dexlansoprazole. (**A**) Counterscreen using His_6_-GST protein was performed to analyze the interference between dexlansoprazole and the assay system. (**B**) Dexlansoprazole identified in precious screening exhibited a sigmoidal dose-dependent reduction of assay signal. (**C**) The structure of dexlansoprazole.

‘Hits’ from the drug screening may become tools for studying the disease, as well as starting points in the hunt for treatments. But many hits are artefacts, as their activity does not depend on a specific, drug-like interaction between molecule and protein^31^. These molecules are previously termed as pan-assay interference compounds (PAINS) or ‘promiscuous inhibitors’ or ‘frequent hitters’^32^,^33^,^34^. The PAINS have defined structures, and mainly cover 10 classes of compounds, such as alkylidene rhodanines, aralkyl pyrroles, cyclopentene-fused tetrahyd roquinolines, alk-ylidene pyrazolidinediones, alkylidene barbiturates and thiobarbiturates, phenolic Mannich bases, hydroxyphenylhydrazones, quinones, catechols and cyanine dye^32^. The structure of dexlansoprazole (Fig. 6C) belonged to neither of current published PAINS, indicating that dexlansoprazole may be a specific inhibitor of PPI.

### Non-specificity counterscreen validation

The false positive compound identified in this HTS may affect the assay by forming hydrogen bonds interaction with His_6_-tag of IN CCD or forming chelating complexes with Ni^2+^-ion covering magnetic beads surface. To verify that dexlansoprazole specifically inhibited the signal of the assay by interfere with PPI itself rather than with the assay system, we used a His_6_-GST protein control that bound to the Ni^2+^ coupling magnetic beads and could be detected by GST antibody, generating an extremely strong signal. Any compound that interfered with bead or with the His_6_-tag of double-tagged protein would result in a decreased signal in the counterscreen assay. As shown in Fig. 6A, dose-response performed in non-specificity counterscreen indicated dexlansoprazole almost had no effect on the assay system, whereas displayed a dose-dependent effect on IN CCD-LEDGF/p75 IBD PPI with an IC_50_ value of 4.8 μM (Fig. 6B).

### Dexlansoprazole directly binds to LEEDGF/p75

**Figure 7:**
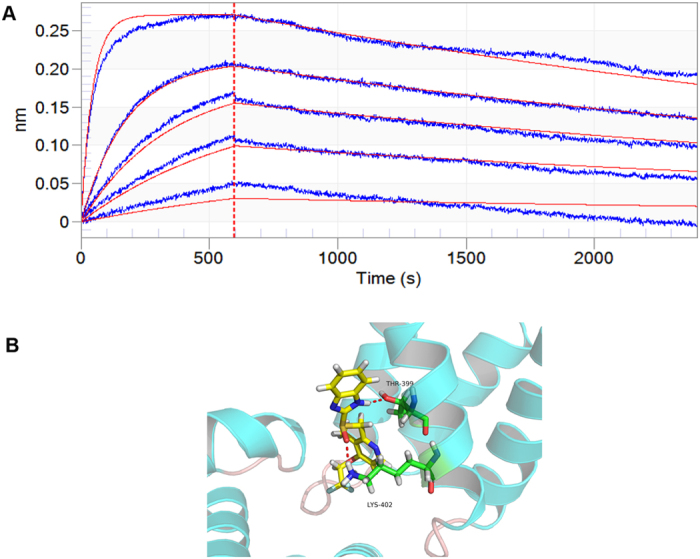
Dexlansoprazole binds directly to LEDGF/p75. (**A**) The BLI results confirmed that dexlansoprazole bound to LEDGF/p75 with a calculated dissociate constant Kd value of 330 nM ± 2.6 nM. The experiments were performed and analyzed using the ForteBIO Octet system. (**B**) Binding mode of dexlansoprazole at the interface of LEDGF/p75-IN interaction (ligand colored in yellow). Red dash lines represent hydrogen bonds interactions between the ligand and receptor.

It is generally believed that molecules targeting the LEDGF/p75 partner of the protein-protein interaction may have the higher genetic barrier to resistance, in contrast to previously reported compounds which block the IN partner^35^,^36^. We therefore utilized biolayer interferometry (BLI) assay to biophysical characterization of dexlansoprazole binding to LEDGF/p75. BLI results demonstrated direct binding of dexlansoprazole to LEDGF/p75 with a calculated dissociation constant Kd value of 330 nM ± 2.6 nM (Fig. 7A). Finally, the binding mode analysis of dexlansoprazole at the PPI interface was performed using PyMOL. The result illustrated in Fig. 7B showed that the binding site amino acid residues which are involved in H-bond interactions with the potent compounds are Thr399 and Lys402 in IBD. All these results indicated that dexlansoprazole was potent small molecular inhibitor for IN CCD-LEDGF/p75 IBD interaction.

Drug repositioning, as a cost effective reduced-risk strategy for developing new drug products, has become a mainstream drug development strategy for major pharmaceutical companies^25^. In this study, we identified dexlansoprazole, a FDA-approved proton pump inhibitor, as IN CCD-LEDGF/p75 interaction inhibitor through a newly developed assay. In a previous drug repositioning screening with cancer xenograft models, dexlansoprazole, as a primary screen hit, was not under further investigation due to its poor anti-cancer activity^37^. Here, we provide the first report, to our knowledge, that dexlansoprazole has a moderate inhibitory activity for IN-LEDGF/p75 interaction. As a matter of fact, in our study other five FDA-approved proton pump inhibitors, such as esomeprazole, pantoprazole, lansoprazole, rabeprazole, omeprazole and tenatoprazole, also displayed weak inhibitory activities against IN CCD-LEDGF/p75 IBD interaction (Table 1) and thus were not be used for further investigation.

In conclusion, we have developed a highly sensitive and effective screening method based on Ni^2+^-NTA coupling magnetic agarose beads to identify inhibitors of IN-LEDGF/p75 interaction, and have successfully identified a FDA-approved drug dexlansoprazole that may prove useful in this regard. Furthermore, dexlansoprazole was proved to be a novel small molecule inhibitor of IN-LEDGF/p75 interaction and has the potential to be a lead compound for further optimization.

## Materials and Methods

### Materials

All general biochemical reagents were obtained from AMRESCO (Solon, USA). Ni-NTA resin and GST resin were purchased from Smart-Lifesciences (Changzhou, China). Ni-NTA Magnetic Agarose Beads were purchased from BEAVER Nano-technologies (Suzhou, China). 96-well nonbinding surface (NBS) microplates were purchased from Corning (New York, USA). Rabbit anti-mouse IgG alkaline phosphatase conjugated antibody and mouse anti-GST antibody were purchased from Sigma (St. Louis, USA). INH5 was synthesized by GL Biochem (Shanghai, China).

### Construction of protein expression plasmids

The full-length human LEDGF/p75 cDNA was chemically synthesized in Genewiz, Inc. (Suzhou, China) and cloned into the *Bam*H I-*Sal* I sites of bacterial protein expression vector pGEX-4T-1 resulting in the recombinant plasmid pGEX-4T-LEDGF/p75. The LEDGF/p75 IBD domain encoding sequence was amplified by PCR and subcloned into the *Bam*H I-*Sal* I sites of the vector pGEX-4T-1, creating pGEX-4T-IBD construct for expression of recombinant N-terminal GST-tagged IBD protein. The recombinant plasmid pGEX-4T-IBD_D366N_ for expression of N-terminal GST–tagged mutation IBD/D366N protein was constructed based on pGEX-4T-IBD using site directed mutagenesis with *Dpn* I. The coding region of the IN CCD (residues 50–212), including the F185K-solubilizing mutation, was PCR amplified from pET28a-IN (conserved in our lab) and subcloned into *Nco* I-*Xho* I sites of the vector pET28a to create the pET28a-CCD for expression of recombinant C-terminal His_6_–tagged CCD protein. The recombinant plasmid pET28a-CCD_W131E_ for expression of C-terminal His_6_-tagged mutation CCD/W131E protein was constructed based on pET28a-CCD using site directed mutagenesis with *Dpn* I. The pGEX-4T-His_6_ plasmid for expression of recombinant C-terminal His_6_-tagged GST (GST-His_6_) used for counterscreen was constructed by inserting the His_6_-tag encoding sequences (CACCACCACCACCACCACTGA) to the *Bam*H I-*Sal* I sites of the vector pGEX-4T-1.

### Recombinant protein expression and purification

LEDGF/p75 IBD, D366N LEDGF/p75 IBD, IN CCD, and W131E IN CCD were expressed and purified as previously described^22^. The double-tagged GST-His_6_ protein was produced using standard conditions as previously described^17^. The concentrations of all proteins were determined by the Bradford assay with bovine serum albumin (BSA) as a standard. 12% SDS-PAGE was performed to determine the purity of proteins.

### His_6_-tag pull-down assay

A His_6_-tag pull down assay was performed as described previously^28^. In brief, His_6_-tagged IN CCD was pre-incubated with LEDGF/p75 IBD in pull down (PD) buffer (25 mM Tris-HCl, pH 7.4, 150 mM NaCl, 20 mM imidazole, 0.1% NP-40, 1 mM MgCl_2_) for 30 min at room temperature (RT). Then, the mixtures were centrifuged for 2 min at 13,000 rpm at 4 °C and supernatants were incubated with 40 μl Ni-NTA-agarose (Novagen, San Diego, CA) and stirred for an additional 30 min at RT. The agarose beads were recovered by centrifugation for 2 min at 500×g at 4 °C and washed three times with 500 μl of PD buffer. The beads binding protein-protein complexes were subjected to SDS-PAGE for separation and visualized by Coomassie Blue stain.

### The IN CCD-LEDGF/p75 IBD PPI assay

The IN CCD-LEDGF/p75 IBD interaction assay was developed based on Ni-NTA Magnetic Agarose Beads (BEAVER Nano-technologies Co., Ltd. Suzhou, China). The assay was optimized for use in 96-well nonbinding surface microplates (Corning, New York, USA) in a final volume of 100 μl per well. Briefly, the assay works as follows. Compounds and proteins were diluted to 10× and 5× working solutions in the 1× assay buffer (25 mM Tris-HCl, pH 7.4, 150 mM NaCl, 1 mM MgCl_2_, 0.05% BSA[v/w]) respectively. Firstly, 10 μl compound dilution, 50 μl 1× buffer, 20 μl each of protein dilutions were pipetted into the wells. The plate was sealed and left to incubate at 200 rpm for 3 h at RT. Secondly, 10 μl of Ni-NTA Magnetic Agarose Beads were added followed by incubating at 900 rpm for 30 min at RT. Thirdly, the wells holding the mixture were placed on a magnetic concentrator, the supernatant was discarded, and the wells were washed 3 times with 200 μl wash buffer (50 mM sodium phosphate, pH 7.4, 150 mM NaCl, 0.005% tween-20[v/v], 20 mM imidazole). Fourthly, the wells were incubated with 100 μl of a 1:4000 solution of mouse anti-GST antibody (Sigma, USA) in antibody dilution buffer (50 mM Sodium Phosphate, pH 7.4, 150 mM NaCl, 0.005% Tween-20[v/v], 0.5%[w/v] BSA) at 900 rpm for 30 min at 37 °C, followed by washing three times. Fifthly, the wells were incubated with 100 μl of a 1:5000 solution of rabbit anti-mouse IgG alkaline phosphatase conjugated antibody (Sigma, USA) in antibody dilution buffer at 900 rpm for 30 min at 37 °C, followed by washing 3 times. Lastly, the color reaction was developed with 100 μl substrate buffer (10 mM diethanolamine, pH 9.8, 0.5 mM MgCl_2_, 8 mM p-NPP) for 30 min at 37 °C. The absorbance (450 nm) of each well was measured in an Enspire multimode reader (PerkinElmer, USA). The reproducibility, signal stability, and robustness of this PPI assay were determined to ensure its compatible for HTS. Furthermore, IL23, an IN CCD-LEDGF/p75 IBD PPI inhibitor, was used to validate the assay efficiency^29^.

### Library screening for inhibitors targeting the IN CCD-LEDGF/p75 IBD PPI

In total, 1000 compounds selected at random from ‘The Spectrum Collection’ were purchased from National compound resource center. The library, which is composed of 2,320 compounds in 10 mM DMSO solution, lists primarily FDA-approved human therapeutic drugs, drug-like compounds, and natural products. Each compound was diluted in DMSO to reach a concentration of 300 μM, which was used as the 10× compound stock^38^. In all screening campaigns, the negative control was based on the use of PPI binding mutant controls (single-point mutation) that would prevent the PPI from forming, and the positive control contained 10% (v/v) DMSO only. The negative and positive control with 8 replicates wells each located randomly among the 96 wells of each plate.

A counterscreen was carried out as described above, with the replacement of purified HIV-1 IN CCD and LEDGF/p75 IBD with 10 nM His_6_-tagged GST protein.

### Biolayer interferometry

Biolayer interferometry (BLI) was used to study the kinetics of dexlansoprazole binding to LEDGF/p75 on a ForteBio Octet, according to the manufacturer’s instructions and published methods. Streptavidin biosensors tips were pre-wetted with buffer and immobilized with biotinylated protein. The biosensors were then transferred into wells containing dexlansoprazole in serial dilutions from 32 μM to 1 μM concentrations. Protein–ligand binding was measured by monitoring the changes in the interferometric profile of the wavelength of light passing through the sensor. Binding curves were analyzed using ForteBIO software, which performs a global fit according to the 1:1 Langmuir model to obtain the kinetic rate constants for each set of interaction conditions.

### Data Analysis

The z′ factor, signal-to-noise ratio (S/N), signal-to-background ratio (S/B), signal window (SW), and coefficient of variation (CV) were calculated and then compared with the minimum pass criteria (z′ >0.5, S/N>10, S/B > 3, SW >2, CV < 20%)^39^,^40^,^41^,^42^. The “hits” for PPI assays were classified as compounds that led to more than 60% disruption of a PPI complex. All HTS data were processed using Excel (Microsoft Corp.) and visualized using Prism 5.0 (GraphPad Software).

## Additional Information

**How to cite this article**: Dawei, Z. *et al.* A novel Assay for Screening Inhibitors Targeting HIV Integrase LEDGF/p75 Interaction Based on Ni^2+^ Coated Magnetic Agarose Beads. *Sci. Rep.* **6**, 33477; doi: 10.1038/srep33477 (2016).

## Figures and Tables

**Table 1:**
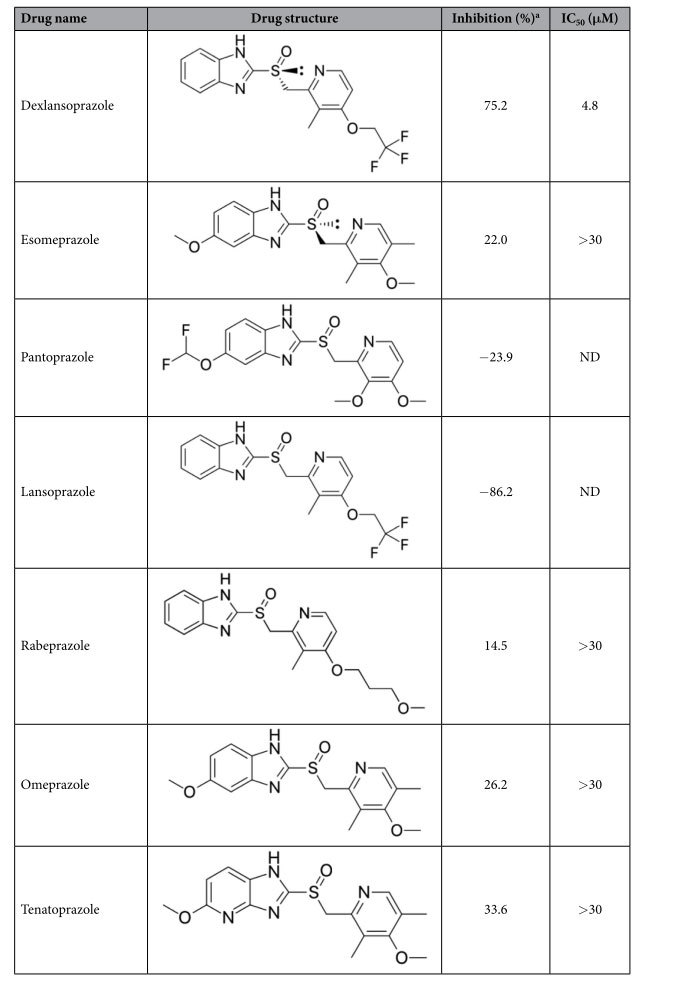
Chemical structures of seven proton-pump inhibitors and their inhibitory data against PPI between LEDGF/p75 IBD and IN CCD.

^a^Data were collected at 30 μM for each proton-pump inhibitor.

## References

[b1] Li X., Krishnan L., Cherepanov P. & Engelman A. Structural biology of retroviral DNA integration. Virology 411, 194–205 (2011). 21216426 10.1016/j.virol.2010.12.008 PMC3640404

[b2] McColl D. J. & Chen X. Strand transfer inhibitors of HIV-1 integrase: bringing IN a new era of antiretroviral therapy. Antiviral. Res. 85, 101–118 (2010). 19925830 10.1016/j.antiviral.2009.11.004

[b3] Hazuda D. J. HIV integrase as a target for antiretroviral therapy. Curr. Opin. HIV AIDS. 7, 383–389 (2012). 22871634 10.1097/COH.0b013e3283567309

[b4] Malet I., Calvez V. & Marcelin A. G. The future of integrase inhibitors of HIV-1. Curr. Opin. Virol. 2, 580–587 (2012). 22980926 10.1016/j.coviro.2012.08.005

[b5] Cooper A. *et al.* HIV-1 causes CD4 cell death through DNA-dependent protein kinase during viral integration. Nature 498, 376–379 (2013). 23739328 10.1038/nature12274

[b6] Skalka A. M. HIV: Integration triggers death. Nature 498, 305–306 (2013). 23739334 10.1038/nature12254

[b7] Steigbigel R. T. *et al.* Raltegravir with optimized background therapy for resistant HIV-1 infection. N. Engl. J. Med. 359, 339–354 (2008). 18650512 10.1056/NEJMoa0708975

[b8] Sichtig N. *et al.* Evolution of raltegravir resistance during therapy. J. Antimicrob. Chemother. 64, 25–32 (2009). 19447792 10.1093/jac/dkp153

[b9] Métifiot M. *et al.* Elvitegravir overcomes resistance to raltegravir induced by integrase mutation Y143. AIDS. 25, 1175–1178 (2011). 21505303 10.1097/QAD.0b013e3283473599 PMC7380719

[b10] Mesplède T. & Wainberg M. A. Is resistance to dolutegravir possible when this drug is used in first-line therapy? Viruses 6, 3377–3385 (2014). 25166745 10.3390/v6093377 PMC4189025

[b11] Van Maele B., Busschots K., Vandekerckhove L., Christ F. & Debyser Z. Cellular co-factors of HIV-1 integration. Trends. Biochem. Sci. 31, 98–105 (2006). 16403635 10.1016/j.tibs.2005.12.002

[b12] Craigie R. & Bushman F. D. HIV DNA integration. Cold Spring Harb. Perspect. Med. 2, a006890 (2012). 22762018 10.1101/cshperspect.a006890 PMC3385939

[b13] Maertens G. *et al.* LEDGF/p75 is essential for nuclear and chromosomal targeting of HIV-1 integrase in human cells. J. Bio. Chem. 278, 33528–33539 (2003). 12796494 10.1074/jbc.M303594200

[b14] Cherepanov P., Ambrosio A. L., Rahman S., Ellenberger T. & Engelman A. Structural basis for the recognition between HIV-1 integrase and transcriptional coactivator p75. Proc. Natl. Acad. Sci. USA. 102, 17308–17313 (2005). 16260736 10.1073/pnas.0506924102 PMC1297672

[b15] Christ F. & Debyser Z. The LEDGF/p75 integrase interaction, a novel target for anti-HIV therapy. Virology 435, 102–109 (2013). 23217620 10.1016/j.virol.2012.09.033

[b16] Debyser Z., Desimmie B. A., Taltynov O., Demeulemeester J. & Christ F. Validation of host factors of HIV integration as novel drug targets for anti-HIV therapy. Med. Chem. Commun. 5, 314–320 (2014).

[b17] Demeulemeester J. *et al.* LEDGINs, non-catalytic site inhibitors of HIV-1 integrase: a patent review (2006–2014). Expert Opin. Ther. Pat. 24, 609–632 (2014). 24666332 10.1517/13543776.2014.898753

[b18] Christ F. *et al.* Rational design of small-molecule inhibitors of the LEDGF/p75-integrase interaction and HIV replication. Nat. chem. Biol. 6, 442–448 (2010). 20473303 10.1038/nchembio.370

[b19] Fader L. D. *et al.* Discovery of BI 224436, a noncatalytic site integrase inhibitor (NCINI) of HIV-1. ACS med. Chem. lett. 5, 422–427 (2014). 24900852 10.1021/ml500002n PMC4027581

[b20] Fenwick C. *et al.* Preclinical profile of BI 224436, a novel HIV-1 non-catalytic-site integrase inhibitor. Antimicrob. Agents Chemother. 58, 3233–3244 (2014). 24663024 10.1128/AAC.02719-13 PMC4068430

[b21] Du L. *et al.* D77, one benzoic acid derivative, functions as a novel anti-HIV-1 inhibitor targeting the interaction between integrase and cellular LEDGF/p75. Biochem. Biophys. Res. Commun. 375, 139–144 (2008). 18691555 10.1016/j.bbrc.2008.07.139

[b22] Hou Y. *et al.* Screening for antiviral inhibitors of the HIV integrase–LEDGF/p75 interaction using the AlphaScreen™ luminescent proximity assay. J. Biomol. Screen. 13, 406–414 (2008). 18480474 10.1177/1087057108317060

[b23] Tsiang M. *et al.* Affinities between the binding partners of the HIV-1 integrase dimer-lens epithelium-derived growth factor (IN dimer-LEDGF) complex. J. Bio. Chem. 284, 33580–33599 (2009). 19801648 10.1074/jbc.M109.040121 PMC2785201

[b24] Jin G. & Wong S. T. Toward better drug repositioning: prioritizing and integrating existing methods into efficient pipelines. Drug Discov. Today. 19, 637–644 (2014). 24239728 10.1016/j.drudis.2013.11.005 PMC4021005

[b25] Smith R. B. Repositioned drugs: integrating intellectual property and regulatory strategies. Drug Discov. Today: Ther. Strateg. 8, 131–137 (2012).

[b26] Tintori C. *et al.* Protein–protein interactions and human cellular cofactors as new targets for HIV therapy. Curr. Opin. Pharmacol. 18, 1–8 (2014). 24993074 10.1016/j.coph.2014.06.005

[b27] Jenkins T. M., Engelman A., Ghirlando R. & Craigie R. A soluble active mutant of HIV-1 integrase involvement of both the core and carboxyl-terminal domains in multimerization. J. Bio. Chem. 271, 7712–7718 (1996). 8631811 10.1074/jbc.271.13.7712

[b28] Busschots K. *et al.* The interaction of LEDGF/p75 with integrase is lentivirus-specific and promotes DNA binding. J. Biol. Chem. 280, 17841–17847 (2005). 15749713 10.1074/jbc.M411681200

[b29] Al-Mawsawi L. Q., Christ F., Dayam R., Debyser Z. & Neamati N. Inhibitory profile of a LEDGF/p75 peptide against HIV-1 integrase: insight into integrase–DNA complex formation and catalysis. FEBS lett. 582, 1425–1430 (2008). 18331842 10.1016/j.febslet.2008.02.076

[b30] Zarzycka B. *et al.* Stabilization of protein–protein interaction complexes through small molecules. Drug Discov. Today 21, 48–57 (2016). 26434617 10.1016/j.drudis.2015.09.011

[b31] Baell J. & Walters M. A. Chemical con artists foil drug discovery. Nature 513, 481–483 (2014). 25254460 10.1038/513481a

[b32] Baell J. B. & Holloway G. A. New substructure filters for removal of pan assay interference compounds (PAINS) from screening libraries and for their exclusion in bioassays. J. Med. Chem. 53, 2719–2740 (2010). 20131845 10.1021/jm901137j

[b33] McGovern S. L., Caselli E., Grigorieff N. & Shoichet B. K. A common mechanism underlying promiscuous inhibitors from virtual and high-throughput screening. J. Med. Chem. 45, 1712–1722 (2002). 11931626 10.1021/jm010533y

[b34] Roche O. *et al.* Development of a virtual screening method for identification of “frequent hitters” in compound libraries. J. Med. Chem. 45, 137–142 (2002). 11754585 10.1021/jm010934d

[b35] Christ F. *et al.* Rational design of small-molecule inhibitors of the LEDGF/p75-integrase interaction and HIV replication. Nat. Chem. Biol. 6, 442–448 (2010). 20473303 10.1038/nchembio.370

[b36] Cavalluzzo C. *et al.* De novo design of small molecule inhibitors targeting the LEDGF/p75-HIV integrase interaction. RSC Advances. 2, 974–984 (2012).

[b37] Roix J. J. *et al.* Systematic repurposing screening in xenograft models identifies approved drugs with novel anti-cancer activity. PLos One. 9, e101708 (2014). 25093583 10.1371/journal.pone.0101708 PMC4122340

[b38] Pereira D. A. & Williams J. A. Origin and evolution of high throughput screening. Br. J. pharmacol. 152, 53–61 (2007). 17603542 10.1038/sj.bjp.0707373 PMC1978279

[b39] Inglese J. *et al.* High-throughput screening assays for the identification of chemical probes. Nat. Chem. Bio. 3, 466–479 (2007). 17637779 10.1038/nchembio.2007.17

[b40] Birmingham A. *et al.* Statistical methods for analysis of high-throughput RNA interference screens. Nat. Methods. 6, 569–575 (2009). 19644458 10.1038/nmeth.1351 PMC2789971

[b41] Zhang J. H., Chung T. D. & Oldenburg K. R. A simple statistical parameter for use in evaluation and validation of high throughput screening assays. J. Biomol. Screen. 4, 67–73 (1999). 10838414 10.1177/108705719900400206

[b42] Schorpp K. *et al.* Identification of small-molecule frequent hitters from AlphaScreen high-throughput screens. J. Biomol. Screen. 19, 715–726 (2014). 24371213 10.1177/1087057113516861 PMC4153540

